# Advances in CRISPR/Cas9-based research related to soybean [*Glycine max* (Linn.) Merr] molecular breeding

**DOI:** 10.3389/fpls.2023.1247707

**Published:** 2023-08-30

**Authors:** Dan Yao, Junming Zhou, Aijing Zhang, Jiaxin Wang, Yixuan Liu, Lixue Wang, Wenxuan Pi, Zihao Li, Wenjun Yue, Jinliang Cai, Huijing Liu, Wenyuan Hao, Xiangchun Qu

**Affiliations:** ^1^ College of Life Science, Jilin Agricultural University, Changchun, Jilin, China; ^2^ Institute of Crop Resources, Jilin Provincial Academy of Agricultural Sciences, Gongzhuling, Jilin, China; ^3^ College of Agronomy, Jilin Agricultural University, Changchun, China; ^4^ Jilin Provincial Academy of Agricultural Sciences, Changchun, Jilin, China

**Keywords:** CRISPR/Cas9, soybean, molecular breeding, gene editing, application

## Abstract

Soybean [*Glycine max* (Linn.) Merr] is a source of plant-based proteins and an essential oilseed crop and industrial raw material. The increase in the demand for soybeans due to societal changes has coincided with the increase in the breeding of soybean varieties with enhanced traits. Earlier gene editing technologies involved zinc finger nucleases and transcription activator-like effector nucleases, but the third-generation gene editing technology uses clustered regularly interspaced short palindromic repeats (CRISPR)/CRISPR-associated protein 9 (Cas9). The rapid development of CRISPR/Cas9 technology has made it one of the most effective, straightforward, affordable, and user-friendly technologies for targeted gene editing. This review summarizes the application of CRISPR/Cas9 technology in soybean molecular breeding. More specifically, it provides an overview of the genes that have been targeted, the type of editing that occurs, the mechanism of action, and the efficiency of gene editing. Furthermore, suggestions for enhancing and accelerating the molecular breeding of novel soybean varieties with ideal traits (e.g., high yield, high quality, and durable disease resistance) are included.

## 1 Introduction

Soybean is a significant source of vegetable proteins for humans and an important oilseed crop, making it a commercially valuable plant (Zhang A, et al., 2023). More than 90% of the soybean plants cultivated in the three main soybean-producing countries (USA, Brazil, and Argentina) are genetically modified varieties generated using gene editing technology (Fang et al., 2023). In terms of sustainable food production, the demand for soybeans has continued to increase because of the scarcity of arable land. In the field of molecular breeding, clustered regularly interspaced short palindromic repeats (CRISPR)/CRISPR-associated protein 9 (Cas9) has emerged as a commonly used third-generation gene editing technology (Nakamori, 2023). Thus, many new and desirable soybean traits have been developed using gene editing technology, which is currently a hot topic in scientific research (Osakabe and Osakabe, 2017; Chen et al., 2022; Zhou et al., 2023a).

In recent years, CRISPR/Cas9 gene editing technology has been used by plant molecular breeders to improve various plant traits (Ma et al., 2016; Zhang et al., 2017; Rao et al., 2022). Because it can simply, effectively, and precisely edit target genes responsible for specific characteristics, CRISPR/Cas9 has replaced previously used gene editing techniques (Zheng et al., 2021; Impens et al., 2022; Liu H. et al., 2022). Several crop traits, including yield, quality, stress tolerance, disease resistance, and herbicide resistance, can be improved using CRISPR/Cas9 systems. This can lead to the development of novel germplasm with superior traits as well as significant advancements in plant molecular breeding (Yin et al., 2017; Hussain et al., 2018; Wada et al., 2020; Gan and Ling, 2022; Qi et al., 2023).

The limitations of early genome editing methods included the inability to explore the relationships between several related genes (Li et al., 2013; Nekrasov et al., 2013; Shan et al., 2013). These previous methods were mostly employed to edit individual genes. Because soybean is a paleotetraploid, it has many homologous and redundant genes, which makes the functional characterization of soybean genes challenging (Tran and Mochida, 2010; Du et al., 2023). The CRISPR/Cas9 system has recently been used to edit multiple genes in the soybean genome. This has considerably decreased the effects of redundant genes on the efficient editing of specific genes for breeding soybean varieties with desirable traits (Bao et al., 2020; Xu H. et al., 2020; Baek et al., 2022; Guan et al., 2022; Rasheed et al., 2022a).

This review describes the recent improvements in soybean traits via the application of the CRISPR/Cas9 gene editing technology. It also presents information regarding the target genes and their mechanism of action, while providing a brief overview of transformation efficiency and gene editing efficiency. Furthermore, suggestions for future CRISPR/Cas9 development and use in soybean molecular breeding programs are included.

## 2 Application of CRISPR/Cas9 gene editing technology in soybean molecular breeding

**Figure 1:**
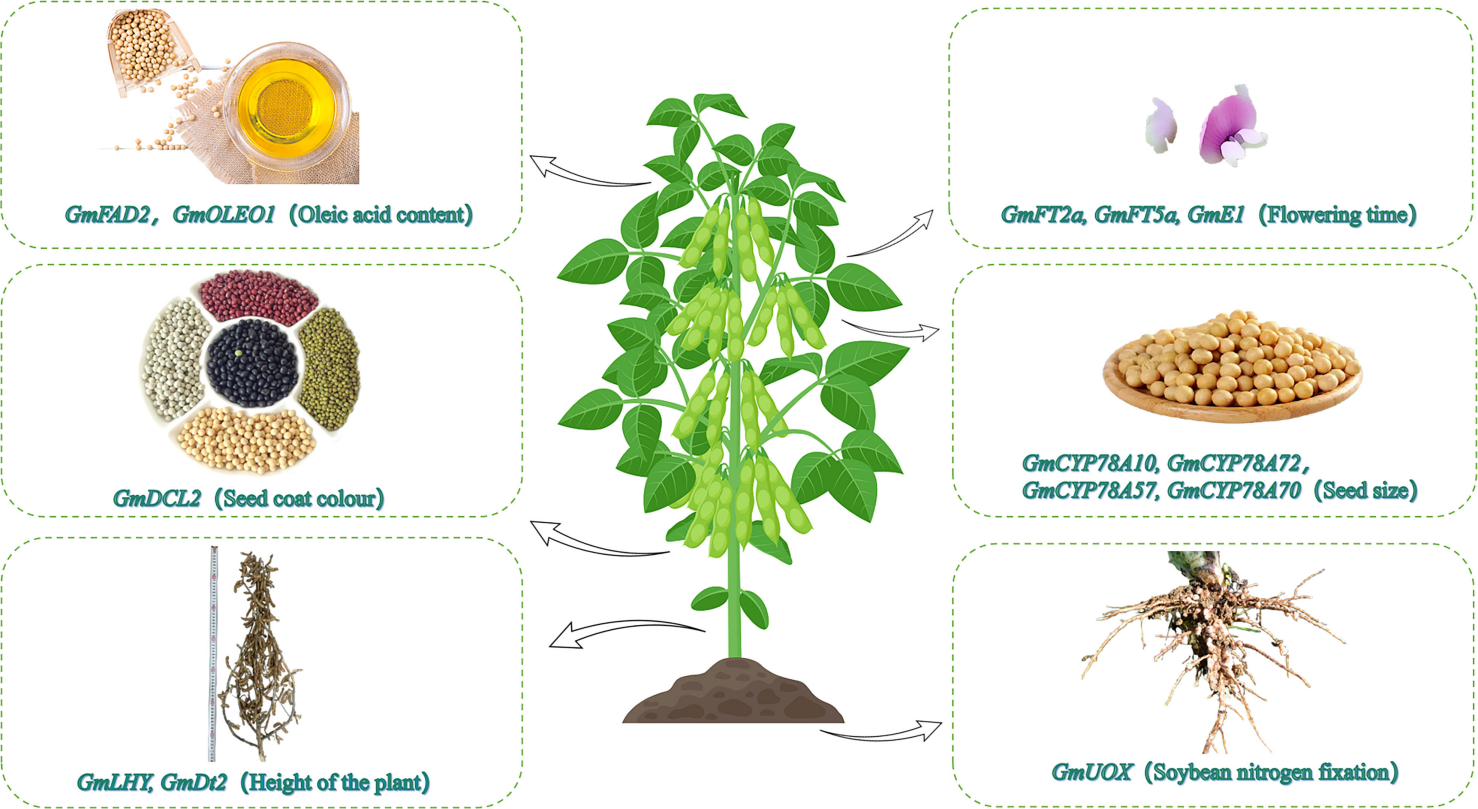
Utility of CRISPR/Cas9 for editing soybean functional genes. The CRISPR/Cas9 gene editing technology has recently been used to modify soybean genes affecting the oil content, photoperiodic flowering, seed coat color, seed size, plant height, and nodulation.

**Figure 2:**
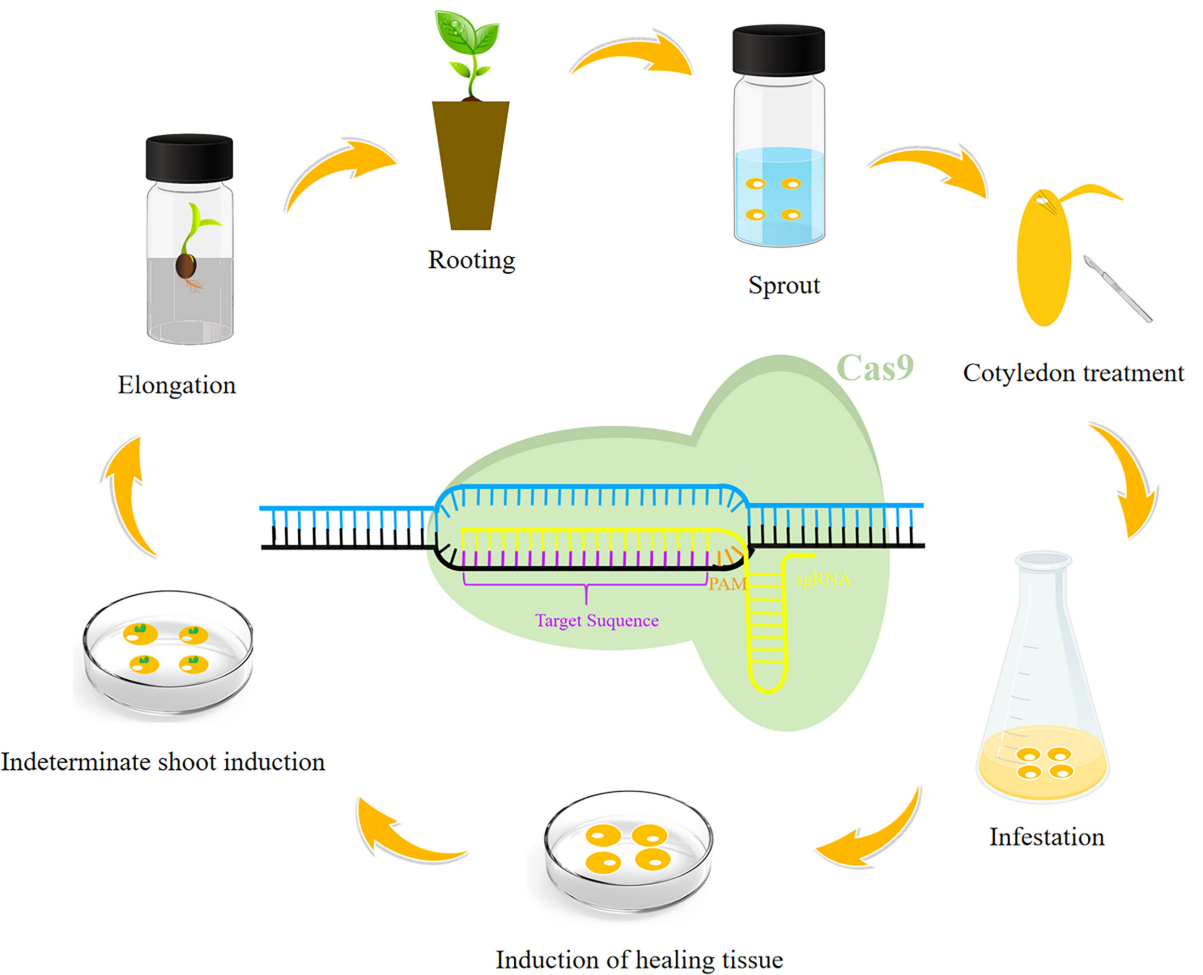
General soybean genetic transformation process. The following steps are generally included in the genetic transformation of soybean: sprouting, cotyledon treatment, infestation, induction of healing tissue, indeterminate shoot induction, elongation, and rooting. A schematic diagram is provided to show how the CRISPR/Cas9 system cleaves the target genomic segment.

There has recently been an increase in the use of CRISPR/Cas9 to edit genes in soybean, corn, wheat, rice, cotton, and other crops (**Figure 1**, **Table 1**). The creation of new soybean germplasm with many excellent traits using various transformation methods (e.g., *Agrobacterium*-mediated transformation) has laid the foundation for further improving CRISPR/Cas9 gene editing technology for soybean molecular breeding (**Figure 2**).

**Table 1:** Applications of CRISPR/Cas9 in five major agricultural crops.

Specie	Gene Name	Gene function	Gene editing method	Edit Type	Editing efficiency	Transformation method	Research significance	Reference
Soybean	*GmFAD2*	Soybean oleic acid content	Single target	Deletion And Insertion	40%-85%	*Agrobacterium*-mediated method	Creation of high oleic acid soybeans	(Zhou et al., 2023b)
	*GmTAP1*	Regulation of soybean resistance to soybean blast	Single target	Deletion And Insertion	Around 50%	*Agrobacterium*-mediated method	Creation of blast-resistant soybean germplasm	(Liu T. F. et al., 2023)
	*GmVPS8a*	Regulation of soybean phenotype	Single target	Deletion	81.25%	*Agrobacterium*-mediated method	Verify that the gene is a multifunctional gene	(Kong et al., 2023)
	*GmPDCT*	Regulation of soybean oil synthesis	Dual Target	Deletion And Insertion	46.7%	*Agrobacterium*-mediated method	Creation of high oleic acid soybean germplasm	(Li et al., 2023b)
	*GmSPL2b*	Regulation of heat tolerance in soybean during flowering	Dual Target	Deletion	–	*Agrobacterium*-mediated method	Creation of heat-resistant soybean varieties	(Ding et al., 2023)
Rice	*Wx/OsBADH9*	Reduced straight-chain starch content and improved aroma	Dual Target	Deletion	Around 55%	*Agrobacterium*-mediated method	Improving the edible quality of hybrid rice	(Tian et al., 2023)
	*OsHPPD*	Herbicide resistance	Single target	Deletion And Insertion	Around 44%	*Agrobacterium*-mediated method	Creation of herbicide-resistant rice	(Wu et al., 2023)
	*OsHPP04*	Anti-parasitic nematode	Dual Target	Deletion And Insertion	Around 30%	*Agrobacterium*-mediated method	Creation of parasitic nematode resistant rice germplasm	(Huang et al., 2023)
	*OsLCD*	Reduction of cadmium accumulation in rice seeds	Dual Target	Deletion And Insertion	–	*Agrobacterium*-mediated method	Creation of low cadmium rice germplasm	(Chen H. M., et al., 2023)
	*OsC1*	Regulation of the phenotype of rice purple leaf sheath	Single target	Deletion	–	*Agrobacterium*-mediated method	Creation of purple sheath deficient phenotype rice germplasm	(Chin et al., 2016)
Maize	*ZmPLA*	Induced haploid germplasm in maize	Triple target	Deletion and Replace	1.04%	Gene gun transformation method	Creation of double haploid germplasm resources of maize	(Rangari et al., 2023)
	*ZmG6PDH1*	Regulation of cold stress tolerance in maize	Dual Target	Deletion	63%-75%	*Agrobacterium*-mediated method	Creation of cold-stress tolerant maize germplasm	(Li et al., 2023a)
	*ZmChSK1*	Regulation of southern leaf blight susceptibility in corn	Dual Target	Deletion And Insertion	13.1%	*Agrobacterium*-mediated method	Creation of southern leaf blight resistant maize germplasm	(Chen C., et al., 2023)
	*ZmbHLH121*	Regulation of cortical gas formation in maize roots	Dual Target	Deletion And Insertion	–	*Agrobacterium*-mediated method	Creation of maize germplasm for elimination of cortical aerial traits in the root system	(Schneider et al., 2023)
	*ZmCals12*	Gene encoding callose synthase	Dual Target	Deletion And Insertion	–	*Agrobacterium*-mediated method	Creation of maize germplasm with male sterile traits	(Niu et al., 2023)
Wheat	*TaTFL1-5*	Regulation of flowering time and inflorescence structure in rice	Single、Dual、 Triple target	Deletion And Insertion	Around 40%	*Agrobacterium*-mediated method	Verification that the regulation of tiller and spikelet formation in wheat has some similar molecular mechanisms	(Sun et al., 2023)
	*TaDCL4、TaDCL5、TaRDR6*	Regulation of male sterility in wheat	Single target	Deletion And Insertion	70%-75%	*Agrobacterium*-mediated method	Creation of male sterile wheat lines	(Zhang R. Z., et al., 2023)
	*TaHRC、Tsn9*	Regulation of disease resistance in wheat	Dual Target	Deletion And Insertion	33%	*Agrobacterium*-mediated method	Creation of wheat germplasm with disease resistance	(Karmacharya et al., 2023)
	*TaPpd*	Regulation of wheat flowering time	Dual Target	Deletion And Insertion	2%	*Agrobacterium*-mediated method	Confirmation that this gene regulates wheat spike structure and grain morphological characteristics	(Errum et al., 2023)
	*TraesFLD1D01G005600、TraesFLD1B01G010600*	Regulating the quality of wheat consumption	Single target	Deletion And Insertion	–	*Agrobacterium*-mediated method	Creation of high quality edible wheat germplasm	(Liu et al., 2023)
Cotton	*GhEMS1*	Regulation of male sterility traits in cotton	Dual Target	Deletion And Insertion	3%	*Agrobacterium*-mediated method	Creation of male sterile cotton germplasm with necrosis-like black spots on anthers	(Zhang J., et al., 2023)
	*GhCLA1*	Regulation of Cotton Whitening Phenotype	Dual Target	Deletion And Insertion	66.7-100%	*Agrobacterium*-mediated method	Achieving multiple gene editing in polyploid crops	(Chen et al., 2021b)
	*GhALARP*	Encodes an alanine-rich protein	Single target	Deletion And Insertion	71.4-100%	*Agrobacterium*-mediated method	Validation of the gene function	(Zhu et al., 2018)
	*GhFAD2*	Regulation of lipid synthesis function	Dual Target	Deletion And Insertion	68.42%-73.68%	*Agrobacterium*-mediated method	Creation of high oleic acid cotton germplasm	(Chen et al., 2021b)
	*GhGPAT12/25*	Regulation of anther cuticle and pollen assembly	Dual Target	Deletion And Insertion	–	*Agrobacterium*-mediated method	Creation of male sterile cotton germplasm	(Zhang et al., 2021)

### 2.1 Enhancement of soybean resistance to abiotic stresses

During different soybean developmental stages, many genetic and biochemical processes control how soybean perceives and responds to abiotic stresses, including salinity and drought. One of the primary objectives of molecular breeding research is improving stress tolerance (Deshmukh et al., 2014; Amoanimaa-Dede et al., 2022; Cadavid et al., 2023). Osmotic stress in plant cells is typically caused by abiotic factors (e.g., drought or excessive salinity). Analyses of the sequences of the related genes revealed the regulatory effects of various plant cellular components, such as sensors, receptors, phytohormones, transcription factors, kinases, phosphatases, and microRNAs, on abiotic stress response-related pathways (Ramesh et al., 2019; Mangena, 2020; Staniak et al., 2023).

Water deficiency substantially restricts soybean growth and development, which can decrease the soybean yield by up to 40% (Khan, 2018). Thus, there is a critical need for exploring the mechanism underlying soybean drought resistance and generating new drought-resistant soybean germplasm (Ramlal et al., 2022). By deleting miR398c in soybean, Zhou et al. (2020) increased the expression of *GmCSD1a/b*, *GmCSD2a/b/c*, and *GmCCS* (relative to the corresponding levels in over-expression strains), thereby increasing the capacity to scavenge O^2−^ (Zhou et al., 2020). In 2021, Xiao et al. identified 112 *GmPLA* family genes in the soybean genome and used CRISPR/Cas9 technology to knock out two homologous genes (*GmpPLA-II* epsilon and zeta). Knocking out one or both genes affected the root response to phosphorus deficiency, with some mutant lines exhibiting increased resistance to flooding and drought conditions (compared with the control) (Xiao et al., 2021). Additionally, in 2021, Yu et al. reported that the *GmNF-YC14* deletion mutant created using CRISPR/Cas9 technology is more susceptible to drought stress than wild-type soybean, implying *GmNF-YC14* may be useful for increasing soybean drought tolerance (Yu et al., 2021). By comparing the agronomic features of soybean plants over-expressing *sHSP26* with those of soybean plants in which *sHSP26* had been edited, Liu S. Y., et al. (2022) revealed that *sHSP26* may considerably increase soybean drought tolerance and yield (Liu S. Y., et al., 2022). In 2022, Yang et al. edited the soybean transcription factor gene *GmNAC12*, which decreased the survival of the transgenic plants exposed to drought stress by at least 12%. They concluded that *GmNAC12* is a key gene that positively regulates soybean tolerance to drought conditions (Yang C.F., et al., 2022).

Salinity can severely decrease the seed yield and quality of soybean, which is a salt-sensitive crop species (Phang et al., 2008; Cai et al., 2022; Feng et al., 2023). In addition to accelerating the development of salt-tolerant soybean varieties to increase grain yield, research on salt stress tolerance can also optimize the use of saline farmland (Chen et al., 2018; Jin et al., 2021). In 2021, Niu et al. clarified the effects of knocking down and over-expressing *lncRNA77580* on the expression of nearby protein-coding genes linked to the soybean response to salt stress. Additionally, increases in the length of the DNA fragment deleted from *lncRNA77580* via the application of CRISPR/Cas9 technology increased the changes in the expression of *lncRNA77580* and nearby genes (Niu et al., 2021). By simultaneously targeting six *GmAITR* genes using a CRISPR/Cas9 system, Wang et al. (2021) produced a Cas9-free *GmAITR3* and *GmAITR6* double mutant and a *GmAITR2 GmAITR3 GmAITR4 GmAITR5 GmAITR6* quintuple mutant. They determined that salt tolerance was more pronounced in the higher-order mutants, suggesting that mutating *GmAITR* genes can enhance soybean salt tolerance (Wang et al., 2021). Zhang M.H. et al. (2022) produced three soybean mutants in which *GmSOS1* was edited and observed that Na^+^ accumulated significantly more in the mutants than in the control. Accordingly, this gene is essential for soybean salt tolerance because it helps maintain Na^+^ homeostasis (Zhang M.H., et al., 2022).

The adaptation of soybean to severe drought and salt stresses involves the activation of overlapping pathways at the morphological, physiological, and molecular levels. Drought tolerance and salt tolerance are polygenic traits (Chen et al., 2018; Kofsky et al., 2018; Mammadov et al., 2018). Additionally, the perception of stress and its effects on soybean growth or development are similar among the abiotic stress factors. In an earlier study by Du et al. (2018), soybean plants in which the transcription factor gene *GmMYB118* was silenced were more susceptible to drought and saline conditions than soybean plants over-expressing *GmMYB118*. Moreover, the decreased production of minor heat shock proteins increased the resistance of plants to drought, cold, and salt stresses (Du et al., 2018). However, when Zhang M.H. et al. (2022) knocked out *GmHsps_p23*, which encodes a minor heat shock protein in soybean, the transgenic plants were highly susceptible to salt and drought conditions. Future research will need to focus on the use of several gene editors to simultaneously target and regulate the expression of functional genes mediating drought and salinity tolerance to produce novel soybean genotypes with superior traits (Zhang Y.Z., et al., 2022).

### 2.2 Enhance disease and insect resistance in soybean

Tobacco ringspot virus, soybean dwarf virus, soybean vein necrosis virus, soybean mosaic virus (SMV), bean pod mottle virus, and alfalfa mosaic virus are only a few of the viruses that can infect soybean (Liu et al., 2016; Widyasari et al., 2020; Lin et al., 2022). Multiple viruses can simultaneously infect soybean plants, causing more harm than an infection by a single virus. Hence, the use of gene editing tools to target genes that control soybean disease resistance and improve disease resistance-related traits has become a major objective in soybean molecular breeding programs (Chang et al., 2016; Chandra et al., 2022; Zhao et al., 2023).

Several non-homologous end-joining and homology directed repair-mediated gene replacement mutants were produced by Fang et al. (2015), who targeted the soybean blast fungal pathogenicity gene *Avr4/6*. These mutants were more resistant to diseases caused by oomycetes than the controls (Fang and Tyler, 2016). Ochola et al. (2020) edited the usual effector genes of the soybean root pathogen *Phytophthora sojae*. They observed that disease resistance was affected by the *Avr* gene expression level in soybean (Ochola et al., 2020). In 2020, Ma et al. confirmed that *GmLMM2* deficiencies increased the resistance to *P. sojae* by increasing tetrapyrrole biosynthesis, but decreased the chlorophyll content by disrupting tetrapyrrole biosynthesis. The elimination of *GmLMM2* expression resulted in the appearance of necrotic regions in the growing leaves of the CRISPR/Cas9-edited mutants (Ma et al., 2020). Zhang P.P, et al. (2020) targeted *GmF3H1*, *GmF3H2*, and *GmFNSII-1* in soybean plants (including the hairy roots) using a CRISPR/Cas9-mediated multiple gene editing system. They detected a significant increase in the isoflavone content and a significant decrease in the SMV coat protein content (approximately 33% decrease) in the mutants, indicating that the increased isoflavone content enhanced the leaf resistance to SMV (Zhang P.P., et al., 2020). Three crucial genes in the soybean *Rsc4* gene family (*Rsc4-1, Rsc4-2, and Rsc4-3*) were modified by CRISPR/Cas9 in 2021 to alter soybean resistance to SMV (Yin et al., 2021). To investigate the effector gene *Avr1b-1* in the soybean pathogen *Blastomyces* in terms of its function as well as the underlying mechanism. Gu et al. (2021) created target locus-specific knockout and knock-in mutants. All selected knockout mutants were virulent on plants expressing *Rps1b*, whereas the infection of plants lacking *Rps1b* was unaffected. When a sgRNA-resistant variant of *Avr1b-1* was re-introduced into the *Avr1b-1* locus of the mutants in which *Avr1b* was knocked out, the resulting knock-in transformants expressing *Avr1b-1* were unable to infect soybean plants carrying *Rps1b* (Gu et al., 2021). Compared with the RNAi and over-expression strains, the soybean plants in which *GmDRR1* was knocked down (in 2022) were considerably less resistant to *Blastomyces* infections (Yu et al., 2022). By altering the coding region of the soybean transcription factor gene *GmTCP19L*, Fan et al. (2022) produced a mutant with a 2 bp deletion. This mutant soybean germplasm resource exhibited increased susceptibility to blast molds (Fan et al., 2022).

Plants that are resistant to *Rps* gene products can perceive certain pathogen effectors encoded by Avr genes. By deleting *Avr45a*, Arsenault-Labrecque et al. (2022) produced novel soybean plants resistant to *Rps8* (Arsenault-Labrecque et al., 2022). In 2022, Zhang et al. identified *Glyma.07g110300* (LOC100775351) as a quantitative trait locus (QTL)-M marker gene encoding the UDP-glycosyltransferase (UGT) primarily responsible for soybean resistance to leaf-chewing insects. Using a CRISPR/Cas9 system, they enhanced the resistance of soybean to *Helicoverpa armigera* and *Spodoptera litura* via the following two mutation types: large fragment deletion and single base insertion. Zhang Y.X., et al. (2022) confirmed that *GmUGT* confers resistance to leaf-chewing insects by changing the flavonoid content and the expression of genes related to flavonoid biosynthesis and defense (Zhang Y.X., et al., 2022). By editing the soybean 14-3-3 gene (*Glyma05g29080*) via large fragment insertions and deletions and producing transgenic plants with increased susceptibility to hard tick infestations and decreased nodulation, Zhang Y.F., et al. (2023) showed *Glyma05g29080* contributes to nodulation and defense responses (Zhang Y.F., et al., 2023). Using a CRISPR/Cas9 gene editing method, Liu et al. (2023b) silenced *GmTAP1* in soybean, which resulted in increased resistance to *P. sojae* strains P231, P233, and P234. An analysis of reactive oxygen species revealed that a loss-of-function mutation to *GmTAP1* does not substantially alter plant basal immunity (Liu T.F., et al., 2023).

The soybean cyst nematode (SCN) is responsible for the soybean disease associated with the largest economic losses (Bent, 2022). By altering two functional genes (*Glyma.12G194800* and *Glyma.16G154200*) in the syntaxin family of SCN resistance genes, Dong et al. (2020) produced SCN-resistant soybean cultivars (Dong et al., 2020). In 2021, Butler et al. demonstrated that *Glyma.15G191200* of cqSCN-006, which encodes gamma-SNAP, influences SCN resistance. Additionally, using CRISPR/Cas9 gene editing technology to disrupt the cqSCN-006 allele decreased the SCN resistance of the transgenic roots (Butler et al., 2021). In 2022, Zhang et al. mutated *Glyma.07g110300* by introducing a CRISPR/Cas9 expression vector into the Tianlong 1 soybean variety to increase the resistance to *S. litura* and *H. armigera* (Zhang Y.X., et al., 2022).

### 2.3 Improvement of seed quality in soybean

Soybean is used as a source of food for animals, including humans (Medic et al., 2014). It has the highest protein content of any crop and is a significant source of edible oils (Gupta and Manjaya, 2022; Zaaboul et al., 2022; Song et al., 2023). In the past few years, several studies have employed CRISPR/Cas9 gene editing technology to enhance the protein and oleic acid contents of soybean.

Using germinal root transformation technology, Li et al. altered the soybean seed storage protein-encoding genes *Glyma.20g148400*, *Glyma.03g163500*, and *Glyma.19g164900* to increase soybean seed protein contents (Li et al., 2019a). By simultaneously modifying the soybean genes *GmFAD2-1A* and *GmFAD2-1B*, Do et al. (2019) managed to increase the oleic acid content by more than 80%, while also decreasing the linoleic acid level by 1.3%–1.7% (Do et al., 2019). Zhang et al. (2019) silenced the soybean phospholipase *D1*-encoding gene, which increased the oil content and germination rate of the mutant seeds (compared with the wild-type seeds) at high temperatures and high humidity levels (Zhang et al., 2019). In 2021, Qu et al. analyzed the oleic acid contents of soybean plants over-expressing *Gm15G117700* and soybean plants in which the gene was edited; the oleic acid content increased in the gene-edited plants by 3.49% (Qu et al., 2021). Zhou et al. (2023a) recently edited five important enzyme-encoding genes in the *GmFAD2* family and analyzed the associated effects on soybean oil synthesis. Editing *GmFAD2-1A* increased the oleic acid content by 91.49% (Zhou et al., 2023a). In another recent study, Li et al. (2023) edited two target genes by altering the conserved PAP2 structural domain-encoding sequences of *GmPDCT1* and *GmPDCT2*. The decrease in phosphatidylcholine-derived diacylglycerol contents via the knockdown of *GmPDCT* prevented the entry of phosphatidylcholine-modified polyunsaturated fatty acids into the triacylglycerol biosynthesis pathway (Li et al., 2023b).

In addition to increasing the protein and oleic acid contents, researchers have attempted to enhance other soybean characteristics. Phytic acid (PA) is an anti-nutrient in grains that prevents humans from absorbing trace minerals (e.g., iron and zinc). In soybean, *GmIPK1* encodes an enzyme that converts inositol 1,3,4,5,6-pentaphosphate to inositol 1,2,3,4,5,6-hexaphosphate (Alkarawi and Zotz, 2014; Sarkhel and Roy, 2022). Using the CRISPR/Cas9 system, Song et al. (2022) edited the *GmIPK1* gene and sgRNA to introduce mutations to create soybean lines with low PA levels. The decreased PA levels in the T_2_ generation mutant seeds were not accompanied by defective growth or seed development (Song et al., 2022).

Flavor is an important soybean quality-related attribute. Accordingly, CRISPR/Cas9 technology has been exploited to develop soybean germplasm with superior flavor-related traits (Fernandez-Marin et al., 2014). Because soybean proteins are allergens, decreasing the abundance of allergenic proteins will likely increase the utility of soybean as a source of protein (e.g., in processed food) (Cordle, 2004; L'Hocine and Boye, 2007; Gharibzahedi et al., 2022; Gracio et al., 2023). In 2020, Sugano et al. simultaneously targeted and edited *GmBd28k* and *GmBd30K* to eliminate two allergenic proteins in the Japanese soybean cultivars Enrei and Kariyutaka (Sugano et al., 2020). Soybean flavor and quality are influenced by three lipoxygenases (LOX1, LOX2, and LOX3). By editing three genes in the soybean *GmLox* family (*GmLox1*, *GmLox2*, and *GmLox3*), Wang J., et al. (2020) improved the edibility of soybean oil and protein products. Editing these genes decreased soybean odors (Wang J., et al., 2020). The raffinose oligosaccharide (RFO) family members are the main soluble carbohydrates in soybean seeds, but they are anti-nutritional seed components because they typically cause gas and indigestion, while also decreasing energy efficiency (Salvi et al., 2022). In 2021, Le et al. decreased the soybean seed RFO content by knocking down two galactinol synthase-encoding genes, namely *GmGOLS1A* and its homolog *GmGOLS1B* (Le et al., 2020). To decrease the RFO content in mature seeds, Cao et al. (2022) used a CRISPR/Cas9 multi-gene editing method to delete the *RS2* and *RS3* genes in soybean and cottonseed (Cao et al., 2022). Qian et al. (2022) mutated *GmBADH2* and confirmed this gene contributes to soybean odors (Qian et al., 2022). In addition, Bai et al. (2022) used CRISPR/Cas9 gene editing technology to produce two multi-gene mutants, one lacking the 7S subunit and the other lacking the 11S subunit. Both of these mutations enhanced the flavor of soybean meal (Bai et al., 2022).

### 2.4 Improvement of phenotype in soybean

One of the key factors influencing the development of high-yielding soybean cultivars is the appropriate regulation of plant structural features (e.g., plant height, number of nodes, number of pods, internode length, number of branches, and number of grains) (Hu and Wiatrak, 2012; Kuzbakova et al., 2022). In recent years, soybean phenotype-related genes have been edited using CRISPR/Cas9 gene editing technology to produce soybean germplasm with a variety of improved features.

Using the CRISPR/Cas9 system, Bao et al. (2019) mutated four *SPL9* family genes that encode *SQUAMOSA* promoter-binding protein-like (SPL) transcription factors. The higher-order mutant plants with different combinations of mutations had more nodes and branches on the main stem (compared with the control plants), resulting in varying numbers of nodes per plant (Bao et al., 2019). In 2019, Cheng et al. used four gRNAs to alter four late elongated hypocotyl (LHY)-encoding *GmLHY* genes in soybean. Phenotypic analyses showed that the quadruple mutant plants had relatively short internodes and exhibited dwarfism (Cheng et al., 2019). In the Tianlong 9 variety, Jia et al. (2020) knocked out two copies of the soybean *DCL2* gene, which altered the color of the soybean seed coat from yellow to brown (Jia et al., 2020). To increase soybean production, Cai et al. (2021) modified the low-latitude spring soybean variety Huachun 6 using a CRISPR/Cas9 multi-gene editing technique. Specifically, they targeted *GmJAG*, which affects the number of seeds per pod (Cai et al., 2021). In 2022, Mu et al. targeted six *GmBIC* genes in soybean using CRISPR/Cas9 technology. The single, double, and quadruple mutants were shorter than normal (Mu et al., 2022). In another recent study, Zhong et al. (2022) edited the soybean *GmHdz4* gene, which increased the total root length, root surface area, and number of root tips (compared with the mutant lines over-expressing *GmHdz4*) (Zhong et al., 2022). Furthermore, Zhang Z. et al. (2023) silenced the soybean *GmNSS* gene, which resulted in the production of abnormally small seeds. (Zhang Z. et al., 2023).

Abscisic acid is an essential phytohormone that controls various processes related to plant growth, development, and stress responses (Nguyen et al., 2023). Using a CRISPR/Cas9 system, Zhang Z. H. et al. (2022) mutated *GmPYL17*, *GmPYL18*, and *GmPYL19*. Compared with the wild-type plants, the mutants were taller, had more branches, and were less sensitive to abscisic acid during the seed germination stage (Zhang Z. H. et al., 2022).

The shattering of soybean pods can significantly decrease yield. By altering the *GmPDH* gene family in soybean variety Huachun 6, Zhang Z. et al. (2022) showed that the *PDH1* mutation dramatically increases pod shatter resistance without modifying other important agronomic parameters (Zhang Z. et al., 2022).

### 2.5 Regulation of nitrogen fixation by nodules

Rhizobia can produce a symbiotic nitrogen-fixation system with legumes that increases plant output without damaging the local ecosystem (Chakraborty et al., 2022; Hawkins and Oresnik, 2022). More than 65% of the nitrogen fixation is due to the symbiotic interaction between rhizobia and legumes (Fields et al., 2021; Jimenez-Guerrero et al., 2022). Soybean converts free nitrogen in the air to chemosynthetic nitrogen that can be absorbed and used by the plant via nitrogen-fixing nodules. This process yields soybean seeds with a high protein content, thereby increasing the nutritional value of soybean (Dadnia, 2011; Meng et al., 2015; Igiehon et al., 2021).

Xu et al. (2021) promoted soybean nodulation by using CRISPR/Cas9 technology to knock down miR9c (Xu et al., 2021). By deleting the soybean *RFG193* gene, Fan et al. (2020) generated transgenic plants with mature nitrogen-fixing nodules on purple or red roots, which produced anthocyanins, whereas nodules were undetectable on the non-transgenic roots (Fan et al., 2020). In 2021, Yang et al. reported that a loss-of-function mutation to *GmHSP17.9* significantly affects soybean plant growth and seed yield through the associated changes to the number of root nodules, nodule fresh weight, nitrogenase activity, poly-hydroxybutyrate vesicles, and urea and total nitrogen contents (Yang Z.W., et al., 2022). Nguyen et al. (2021) silenced *GmUOX* in a soybean mutant, which exhibited nitrogen deficit atrophy and early nodule senescence as revealed by decreased nitrogenase (acetylene reduction) activities in the nodules, a greenish-white hue inside the nodules, and a decreased root protein output (Nguyen et al., 2021). Gao et al. (2021) investigated the role of the PIN protein during the nitrogen fixation by soybean nodules. More specifically, they produced a triple mutant (*GmPIN1*-abc family) (Gao et al., 2021). The modification of the soybean *Rfg1* allele by Fan et al. (2022) revealed *Rfg1* mediates the resistance to *Sinorhizobium fredii* and *Bradyrhizobium japonicum* strains, leading to broad-spectrum resistance to nodulation in transgenic plants (Fan et al., 2017). After knocking down *GmNN1*, Li et al. (2022) detected yellowing leaves as well as decreased nitrogen contents and decreased nodulation (compared with the wild-type control plants) (Li et al., 2022). By silencing *GmNAC039* and *GmNAC018* as well as the four target genes *GmCYP35*, *GmCYP37*, *GmCYP39*, and *GmCYP4*, Yu et al. (2023) showed that the transcription factors encoded by *GmNAC039* and *GmNAC018* directly increase the expression of *GmCYP* genes to induce root tumor senescence (Yu et al., 2023).

### 2.6 Regulation of flowering time in soybean

Because soybean is a short-day (SD) plant, it blooms more quickly during SD conditions than during long-day (LD) conditions (Weller and Ortega, 2015; Lin et al., 2021; Xia et al., 2021). Modulating the blooming time and minimizing the sensitivity to sunshine duration through molecular breeding can increase soybean adaptability and production by mitigating photoperiodic responses (Zhang L.X. et al., 2020; Zhang M. et al., 2022; Du et al., 2023).

Cai et al. (2018a) edited the soybean genes *GmFT2a* and *GmFT9a* and discovered that both mutants in the T_2_ generation exhibited a late-blooming phenotype (Cai et al., 2018a). Using a double sgRNA design and CRISPR/Cas9 technology, Cai et al. (2018b) deleted specific DNA fragments in *GmFT2a (Glyma16g26660)* and *GmFT5a (Glyma16g04830)*. The homozygous *GmFT2a* mutants (1,618 bp deletion) in the T_2_ generation flowered late (Cai et al., 2018b). Two QTL regions that respectively included *GmFT2a* and *GmFT5a* were identified by Cai et al. (2020b). They were linked to various genetic effects on flowering during various photoperiods. Under LD and SD conditions, the flowering times of transgenic plants over-expressing *GmFT2a* or *GmFT5a*, *GmFT2a* mutants, *GmFT5a* mutants, and *GmFT2a* and *GmFT5a* double mutants were examined. There was no overlap between *GmFT2a* and *GmFT5a*, which cooperatively control the blooming time, but *GmFT2a* has a greater effect than *GmFT5a* under SD conditions, while *GmFT5a* has a greater effect than *GmFT2a* under LD conditions (Cai et al., 2020a). Wang L. W. et al. (2020) mapped QTLs and identified *GmPRR37* as a functional gene encoding a regulator of soybean flowering. A natural mutation to *GmPRR37* results in early flowering, thereby enabling the cultivation of soybean plants at high latitudes (Wang L. W., et al., 2020). Li et al. (2020) used CRISPR/Cas9 technology to knock out *GmPRR3b*. The resulting soybean mutant exhibited retarded growth and a delayed transition to the flowering stage (Li et al., 2020). In 2020, Chen et al. modified the soybean *GmAP1* gene in a quadruple mutant. The observed increase in plant height was associated with delayed flowering, altered flower shapes, and increases in the number of nodes and the internode length. In contrast, under SD conditions, the over-expression of *GmAP1* led to early flowering and dwarfism (Chen et al., 2020). Li et al. (2021) edited four *LNK2* genes using a CRISPR/Cas9 system to produce a quadruple mutant lacking transgenes. This mutant flowered earlier than the wild-type control under LD conditions. In addition, the *LNK2* transcript level was lower in the quadruple mutant than in the wild-type plants (Li et al., 2021). Zhao et al. (2022) mutated *GmPHYA* or *GmPHYB* using CRISPR/Cas9 technology. The phenotypic changes due to the mutations to *GmPHYA2* and *GmPHYA3*, which have redundant and additive roles in seedling responses to daylight, indicated *GmPHYB1* is primarily responsible for daylight-induced photomorphogenesis (Zhao et al., 2022). In 2022, Zhai et al. suggested that *GmMDE* and *GmFT2a*/*GmFT5a* contribute to a positive feedback regulatory loop that promotes flowering in soybean. Knocking down the soybean *E1* gene induces *GmMDE* expression. Moreover, the over-expression of *GmMDE06* increases the expression of *GmFT2a* and *GmFT5a*, which regulate flowering (Zhai et al., 2022). In 2023, Wan et al. investigated the relationship between the dominant *E1* gene and photoperiodic regulation via the CRISPR/Cas9-mediated targeted mutation of *E1* in soybean variety Tianlong 1. Four mutations were introduced into the *E1* coding region. The significant structural changes in the generated mutants included the commencement of terminal flowering, the creation of distinct stems, and a decrease in the number of branches (Wan et al., 2022).

### 2.7 Creation of male sterile soybean germplasm resources

Because soybean is a self-pollinated plant that has small flower organs, artificial cross-breeding is both difficult and ineffective (Li et al., 2019b; Chen G. M., et al., 2021). Furthermore, differences in flowering times among varieties originating from various geographical regions frequently further restrict the exchange of genes, resulting in a limited genetic base for soybean breeding and genetic modifications (Li et al., 2019b). Accordingly, methods for increasing the genetic diversity of soybean varieties are needed (Bohra et al., 2016). In particular, for sexually reproducing crops, male sterility is a crucial precondition for hybrid seed generation and crop reproduction (Jiang et al., 2011; Yang et al., 2014). Male sterile lines can increase the quality of hybrids, lower the cost of hybrid seed production, and even broaden the utility of hybrids. The scarcity of adequate male sterile lines has limited the commercial use of soybean accessions (Li et al., 2016; Ramlal et al., 2022).

To create stable male sterile soybean lines, Chen et al. (2021) targeted *AMS* homologs using CRISPR/Cas9 technology. Although editing *GmAMS2* failed to produce a male sterile line, editing *GmAMS1* yielded plants with a male sterile phenotype. *GmAMS1* contributes to the development of pollen walls as well as the regulation of soybean tapetum degeneration (Chen et al., 2021a). Jiang et al. (2021) modified *Glyma.13G114200* using a CRISPR/Cas9 system; the phenotypes of two gene-edited lines were consistent with the male sterility of the *MS1* mutant (Jiang et al., 2021). By eliminating *GmSPL2b*, Ding et al. (2023) decreased the heat tolerance of a soybean cytoplasmic male sterility-based recovery line during flowering (Ding et al., 2023).

### 2.8 Application of other CRISPR gene editing technology in soybean

Compared with Cas9, the CRISPR family member Cas12a is more practical and effective. Hence, CRISPR/Cas12a can effectively edit multiple genes because of the specific way that CRISPR RNA (crRNA) functions (Bandyopadhyay et al., 2020; Paul and Montoya, 2020; Zhou et al., 2023b). In 2017, Jiang et al. used CRISPR/Cas12a to achieve editing in the soybean *FAD2* gene for the first time (Jiang et al., 2017). In addition, large chromosomal segments of the target genome were deleted by Duan et al. (2021) using CRISPR/Cas12a, with an editing efficiency of 91.7% (Duan et al., 2021). In 2023, Liang et al. produced CRISPR/Cas12a-edited soybeans in just 45 days, with transformation and gene editing efficiencies of 30% and 50%, respectively (Liang et al., 2023). To produce gene-edited soybeans with better traits, CRISPR/Cas12a-based multi-gene editing methods will increasingly be used to modify the soybean genome.

Because they enable the replacement of a single base via RNA editing without introducing DNA double-strand breaks or requiring donor templates, base editor tools created using the CRISPR/Cas9 system are especially useful for plant molecular breeding (Molla et al., 2021; Yang et al., 2021; Hua et al., 2022). A CRISPR/Cas9-mediated base editing tool was designed by Cai et al. (2020a) to alter individual bases in the soybean genome. A base editor was developed by combining Cas9n (D10A), rat cytosine deaminase (APOBEC1), and a uracil glycosylase inhibitor. This base editor was then cloned into the pTF101.1 vector. The targeted genes were *GmFT2a* and *GmFT4a*, which were under the control of the 2× CaMV 35S promoter. There were two types of base substitutions (C to T and C to G), both of which occurred within the target sequence (Cai et al., 2020a). Single nucleotide polymorphisms, which influence phenotypic diversity and are linked to many significant agronomic parameters, are abundant in the soybean genome. Future genetic improvement and breeding of soybean can greatly benefit from the application of base editing technology (Bharat et al., 2020; Xu R. F., et al., 2020).

## 3 Discussion and prospect

Because of increases in the global population and living standards, CRISPR/Cas9 technology must be exploited to quickly develop high-yielding, high-quality soybean varieties (Khan et al., 2018; Zhang and Showalter, 2020). Field tests of high-oleic soybean varieties produced using CRISPR/Cas9 gene editing technology in the US have produced positive results, with potential implications for soybean molecular breeding. There have been considerable advances in the molecular breeding of soybean since the development of CRISPR/Cas9 gene editing technology, which has decreased concerns about the safety of products made from genetically modified soybeans, leading to the gradual acceptance of genetically modified crops. The CRISPR/Cas9 system, which continues to be refined and enhanced, has largely outperformed the older technologies involving zinc finger nucleases and transcription activator-like effector nucleases in terms of gene editing efficiency and convenience (Samanta et al., 2016; Demirci et al., 2018; Farooq et al., 2018). Researchers will use CRISPR/Cas9 gene editing systems to develop soybean lines with improved features as more functional soybean genes are identified and characterized.

However, there are certain limitations to the utility of CRISPR/Cas9 for soybean breeding. Unanswered questions include the following: (i) How can genome editing tools be efficiently delivered to soybean plants? (ii) How can the functional redundancy in gene families be rapidly and precisely determined? (iii) How can the editing of multiple genes be exploited to modify various traits? (iv) How can base editing, prime editing, and government regulations regarding genome-edited crops further increase the effectiveness of gene editing? Despite encouraging results, many obstacles must be overcome before CRISPR/Cas9 can be widely used for soybean breeding.

Additionally, numerous sgRNAs for different plant genomes have been assembled into CRISPR editing vectors. Moreover, sgRNA pooling techniques have made it possible to mutate multiple genes. The diversity in the sequences that PAM can detect has increased, leading to improved gene editing, because of the creation of Cas9 homologs, such as StCas9 and SaCas9, for plant molecular breeding. The highly efficient editing of plant genomes has been achieved using the nCas9-mediated single-base editing system, while the saturation mutagenesis of plant genomes and optimal gene editing efficiencies have been attained via the two-base editing method. The CRISPR/Cas9 gene editing method will be applied to soybean molecular breeding more effectively, conveniently, and broadly in the future, thereby facilitating increasingly precise molecular breeding and accelerating soybean molecular breeding.

## Author contributions

DY and JZ performed the manuscript writing; AZ, JW, YL, LW, WP, ZL summarized the literature reports; WY and JC carried out the production of pictures; HL performed the organization of the table; WH and XQ reviewed and proofread the manuscript. All authors reviewed the manuscript. All authors contributed to the article and approved the submitted version.
